# SIRT1 Is a Regulator in High Glucose-Induced Inflammatory Response in RAW264.7 Cells

**DOI:** 10.1371/journal.pone.0120849

**Published:** 2015-03-20

**Authors:** Yanhui Jia, Zhao Zheng, Yunchuan Wang, Qin Zhou, Weixia Cai, Wenbin Jia, Longlong Yang, Maolong Dong, Xiongxiang Zhu, Linlin Su, Dahai Hu

**Affiliations:** Department of Burns and Cutaneous Surgery, Xijing Hospital, Fourth Military Medical University, Xi’an, Shaanxi, China

## Abstract

Sepsis is defined as a systemic inflammatory response syndrome that disorders the functions of host immune system, including the imbalance between pro- and anti-inflammatory responses mediated by immune macrophages. Sepsis could also induce acute hyperglycemia. Studies have shown that the silent mating type information regulation 2 homolog 1 (SIRT1), an NAD^+^-dependent deacetylase, mediates NF-κb deacetylation and inhibits its function. Therefore, SIRT1 is likely to play an important role in high glucose-mediated inflammatory signalings. Here we demonstrate that high glucose significantly downregulates both the mRNA and protein levels of SIRT1 and upregulates the mRNA level and the release of two pro-inflammatory cytokines, IL-1β and TNF-α, in RAW264.7 macrophages. Interestingly, the reduced level of SIRT1 by high glucose is remarkably upregulated by SIRT1 activator SRT1720, while the level and the release of IL-1β and TNF-α significantly decrease with the use of SRT1720. However, when the function of SIRT1 is inhibited by EX527 or its expression is suppressed by RNAi, the upregulated level and release of IL-1β and TNF-α by high glucose are further increased. Taken together, these findings collectively suggest that SIRT1 is an important regulator in many high glucose-related inflammatory diseases such as sepsis.

## Introduction

Sepsis is defined as a systemic inflammatory response syndrome mediated by a harmful host immune response to infection. Lipopolysaccharide (LPS), a component of the outer membrane of gram-negative bacteria, is a common cause of sepsis via various immune cells, including monocytes and macrophages [1,2]. Sepsis is known to induce acute hyperglycemia [3,4], and its progression is usually accompanied with the change of glycemia levels. Especially, the concentration of glucose has shown to accelerate the aggravation of sepsis [3,5].

Macrophages are important cells involved in inflammation, which have been implicated in the initiation of inflammatory response and play critical roles in the pathogenesis of numerous inflammatory diseases by secreting various inflammatory mediators/cytokines. The nuclear factor kappa B (NF-κB), a proinflammatory transcription factor, is a heterodimer composed of p50 and RelA/p65 subunits and is a key mediator of the immune response in macrophages [6]. In unstimulated cells, NF-κB resides in the cytoplasm bound to inhibitory proteins of the inhibitor of κB (IκB) family [7,8]. Stimulation of cells by environmental factors liberates NF-κB, allowing it to translocate to the nucleus, where it further mediates the transcription of targeting genes, including IL-1β and TNF-α.

The mammalian sirtuin members, named SIRT1 to SIRT7, are highly conserved NAD^+^-dependent deacetylases that have emerged as key factors involved in aging and metabolism, including to adapt gene expression and metabolism to the cellular energy state [9,10]. SIRT1, the leading family member, has been reported to promote longevity in species ranging from yeast to flies [11,12]. SIRT1 is a known suppressor of NF-κB activity, it deacetylates the NF-κB subunit RelA/p65 at lysine310 and thereby inhibits transcription[7,13].

Our study aims at examining the effect of high glucose on the expression levels of SIRT1 and related proinflammatory cytokines in RAW264.7 macrophages, and also to investigate the potential role of SIRT1 in inflammatory response with the use of SIRT1 activator, inhibitor or SIRT1 siRNA.

## Materials and Methods

### Cell Cultures

The immortal mouse macrophage cell line RAW264.7 was obtained from American Type Culture Collection (Livingstone, MT). Cells were cultured in Dulbecco’s modified Eagle’s medium (DMEM; Gibco, Gaithersburg, MD) containing 10% fetal bovine serum (Gibco), 100 IU/ml penicillin and 100 mg/ml streptomycin (Beyotime, Shanghai, China), and maintained at 37°C in a humidified incubator with 5% CO_2_. To harvest RAW264.7 cells, they were first trypsinized with 0.25% trypsin/EDTA in phosphate-buffered saline (PBS) and partly collected, then the remaining undetached cells were further collected by scraping. Cells were then centrifuged at 400 g for 5 min, and then resuspended in serum-free DMEM. Cells were seeded in six-well plates (approximately 3.0 × 10^4^ cells/cm^2^) before further treatments.

### Western Blot

Cells were washed twice with precooled PBS and then lysed in RIPA buffer (50 mM Tris-HCl/pH 7.4, 150 mM NaCl, 1 mM EDTA, 1 mM EGTA, 1% Triton X-100, 1% sodium deoxycholate and 0.1% SDS). The protein concentration was measured using the BCA Protein Assay Kit (Biyotime). 50 μg of total protein extracts were resolved by SDS-PAGE and transfered onto PVDF membrane. The membrane was blocked with 5% non-fat milk and incubated with mouse anti-SIRT1 monoclonal antibody (Catalog# ab110304, clone# 19A7AB4; Abcam, Cambridge, UK) or rabbit anti-β-ACTIN polyclonal antibody (Catalog# CW0097; Cwbiotech, Beijing, China) overnight at 4°C. The next day, the membrane was washed with 1 × TBST (13.7 mM NaCl, 0.27 mM KCl, 2.5 mM Tris, 0.1% Tween-20, pH 7.8) and then incubated with horseradish peroxidase (HRP)-conjugated goat anti-mouse IgG or goat anti-rabbit IgG (Cwbiotech) for 1 h at room temperature. Immunoreactive proteins were detected using Chemiluminescent HRP Substrate (Millipore, Billerica, MA). Bands were quantitated by ImageJ software and the fold expression was indicated as the relative protein level.

### Cell Cytotoxicity Assay

Cells were seeded in 96-well dishes at a density of 3.0 × 10^3^ cells/cm^2^ and treated with high glucose at the concentrations of 5.6, 11.1, 25 and 30 mM, alone or with SRT1720 (1 μM; Catalog# 1001645–58–4; Selleck, Houston, TX) or EX527(10 μM; Catalog# 49843–98–3; Cayman Chemical, Ann Arbor, MI) for 24 h. The stock solution of SRT1720 or EX527 was prepared by dissolving each of them (in powder form) respectively in DMSO yielding a concentration of 100 μM and then stored at -80°C. MTT solution (0.5 mg/ml) was then added to each well and cells were incubated for 4 h at 37°C in a 5% CO_2_ incubator. Subsequently, the supernatant was removed, the formation of farmazan was solubilized with dimethyl sulfoxide (DMSO) and measured at 540 nm with a Bio-Rad Model 680 Plate Reader (Bio-Rad Laboratories, Hercules, CA).

### Enzyme-Linked Immunosorbent Assay (ELISA)

Supernatants from RAW264.7 macrophage cultures were harvested at indicated time points. Mouse TNF-α and mouse IL-1β were detected using ELISA kit (RayBio, GA, USA; NeoBioscience, Shenzhen, China) respectively according to the manufacturer’s instructions. Concentration was calculated by regression analysis of a standard curve.

### Real-time PCR

Total RNA was extracted from RAW264.7 cells by using RisoPlus (TaKaRa, Shiga, Japan) according to the manufacturer’s protocol. Obtained RNA was reverse transcribed into cDNA using the PrimeScript RT Master Mix Kit (Perfect Real Time) (TaKaRa). The primer sequences for each gene were listed as following:

*SIRT1*: Sense, 5'-CAGACCCTCAAGCCATGTTTGATA-3'; Anti-sense, 5'-TTGGATTCCTGCAACCTGCTC-3'. *TNF-α*: Sense, 5'-CGTCAGCCGATTTGCTATCT-3'; Anti-sense, 5'-CGGACTCCGCAAAGTCTAAG-3'. *IL-1β*: Sense, 5'-GCCCATCCTCTGTGACTCAT-3'; Anti-sense, 5'-AGGCCACAGGTATTTTGTCG-3'. *GAPDH*: Sense, 5'-TGTGTCCGTCGTGGATCTGA-3'; Anti-sense, 5'-TTGCTGTTGAAGTCGCAGGAG-3'.

### RNA Interference

The siRNA duplexes for SIRT1 or scramble control were purchased from GenePharma (Shanghai, China), sequences were listed as following:

*Sirt1-siRNA-2195*: Sense, 5'-GGGAUCAAGAGGUUGUUAATT-3'; Anti-sense, 5'-UUAACAACCUCUUGAUCCCTT-3'. *Sirt1-siRNA-1003*: Sense, 5'-CCGUCUCUGUGUCACAAAUTT-3'; Anti-sense, 5'-AUUUGUGACACAGAGACGGTT-3'. *Sirt1-siRNA-576*: Sense, 5'-GCGGAUAGGUCCAUAUACUTT-3'; Anti-sense, 5'-AGUAUAUGGACCUAUCCGCTT-3'. *Scramble siRNA*: Sense, 5'-UUCUCCGAACGUGUCACGUTT-3'; Anti-sense, 5'-ACGUGACACGUUCGGAGAATT-3'.

siRNAs were transfected into RAW264.7 cells with Lipofectamine 2000 Transfection Reagent (Invitrogen, Carlsbad, CA) according to the manufacturer’s instructions. The culture medium containing the transfection mixture was removed 6 h later and repleaced with fresh miedium. The transfected cells were continued to be incubated at 37°C in a 5% CO_2_ incubator for another 42 h and used for further assays.

### Statistical Analysis

All experiments were performed at least three times using different batches of cells. Results were presented as mean ± SEM. Statistical analysis of the data was performed using SPSS17.0 software (SPSS, Chicago, IL) by one-way analysis of variance (ANOVA) with LSD’s or S-N-K’s post-hoc analysis. Values of *p* < 0.05 were considered to be statistically significant.

## Results

### High Glucose Mitigates SIRT1 Levels in RAW264.7 Cells without Comprising Cell Viability

**Fig 1:**
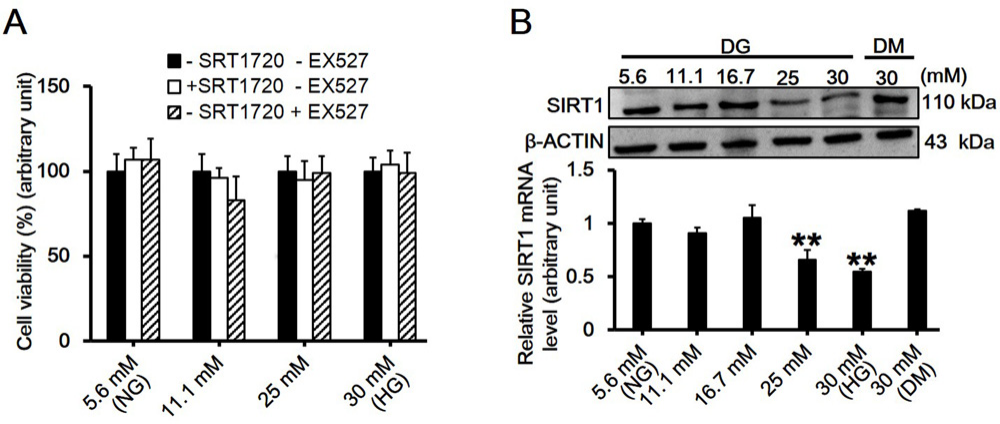
Assessment of the effects of high glucose on cell viability and SIRT1 expression in RAW264.7 cells. (A) RAW264.7 cells were treated with D-glucose at gradually increased concentrations of 5.6, 11.1, 25 and 30 mM at the presence of SRT1720 (1 μM, SIRT1 activator) or EX527 (10 μM, SIRT1 inhibitor) for 24h, and the cell viability was evaluated by MTT assay. Each data point is the mean ± SEM of *n* = 3 with the value in 5.6 mM D-glucose without SRT1720 and EX527 group arbitrarily set as 100%. (B) The expression levels of SIRT1 in RAW264.7 cells treated with different concentrations of D-glucose (DG) at 5.6, 11.1, 16.7, 25 and 30 mM for 24h were evaluated by western blot. 30 mM of D-mannitol (DM) served as an osmotic control. Each data point is the mean ± SEM of *n* = 3 and normalized against corresponding β-ACTIN protein level with the value in 5.6 mM group arbitrarily set as 1 (*df* = 5, *f* = 15.3, *p* = 0.002). **p < 0.01. ‘NG’, normal glucose, standing for 5.6 mM D-glucose throughout the whole study; ‘HG’, high glucose, standing for 30 mM D-glucose throughout the whole study.

To determine the cytotoxicity of high glucose, alone or with its activator SRT1720 or inhibitor EX527, MTT assay was performed. Results showed that neither high glucose up to 30 mM nor SRT1720 or EX527 reduced the viability of RAW264.7 macrophage cells (Fig. 1A). The protein level of SIRT1 in RAW264.7 cells treated with different concentrations of high glucose was assessed by immunobloting. Results showed that *in vitro* stimulation of RAW264.7 macrophages submitted to glucose at various concentrations, and 30 mM high glucose exerted the maximum suppressive effect on SIRT1 level (Fig. 1B; *df* = 5, *f* = 15.3, *p* = 0.002).

### High Glucose Transiently Downregulates Both the mRNA and Protein Levels of SIRT1 in RAW264.7 Cells

**Fig 2:**
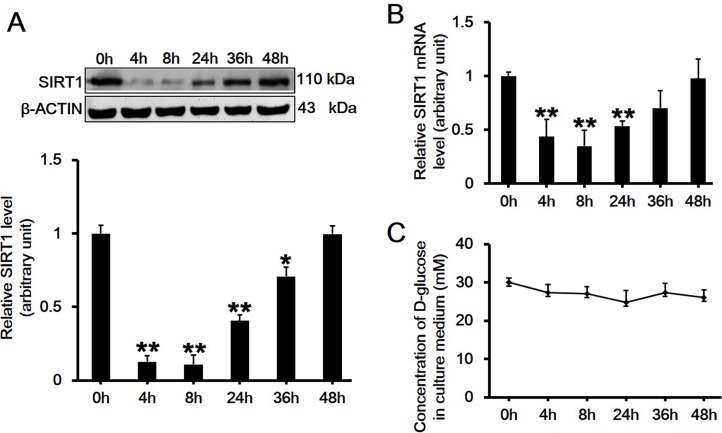
Assessment of the protein and mRNA level changes of SIRT1 in RAW264.7 cells treated with 30 mM high glucose over time. (A-B) Immunoblotting (A) or quantatitive RT-PCR (B) assessing the change in SIRT1 protein or mRNA level after treatment of RAW264.7 cells with 30 mM high glucose at indicated time points (0, 4, 8, 24, 36 and 48h), respectively. Each data point is the mean ± SEM of *n* = 3 and normalized against corresponding β-ACTIN protein level (A, *df* = 5, *f* = 134.651, *p* < 0.001) or *GAPDH* mRNA level (B, *df* = 5, *f* = 127.339, *p* < 0.001) with the value at 0h arbitrarily set as 1. *p < 0.05; **p < 0.01. (C) The concentration of D-glucose in the culture medium was measured over time using ONETOUCH Ultra at indicated time points (0, 4, 8, 24, 36 and 48h). Each data point is the mean ± SEM of *n* = 3.

RAW264.7 cells were treated with 30 mM D-glucose for different time periods, then the protein or mRNA level of SIRT1 was assessed by western blot or quantitative real-time PCR, respectively. Results showed that both the protein (Fig. 2A; *df* = 5, *f* = 134.651, *p* < 0.001) and mRNA levels (Fig. 2B; *df* = 5, *f* = 127.339, *p* < 0.001) of SIRT1 showed significant transient downregulation after 30 mM high glucose treatment. The maximum suppressive effect appeared between 4 to 8 h, and then the SIRT1 level gradually increased from 24 h and showed no difference at 48 h post-treatment (Fig. 2A-B). To determine whether the level change of SIRT1 was associated with D-glucose consumption in the culture medium, we then measured the glucose concentration in the supernatant at selected time points. Results showed that the concentration of D-glucose in culture medium did not change over time (Fig. 2C).

### High Glucose Induces the Upregulation of the mRNA levels of IL-1β and TNF-α in RAW264.7 Cells

**Fig 3:**
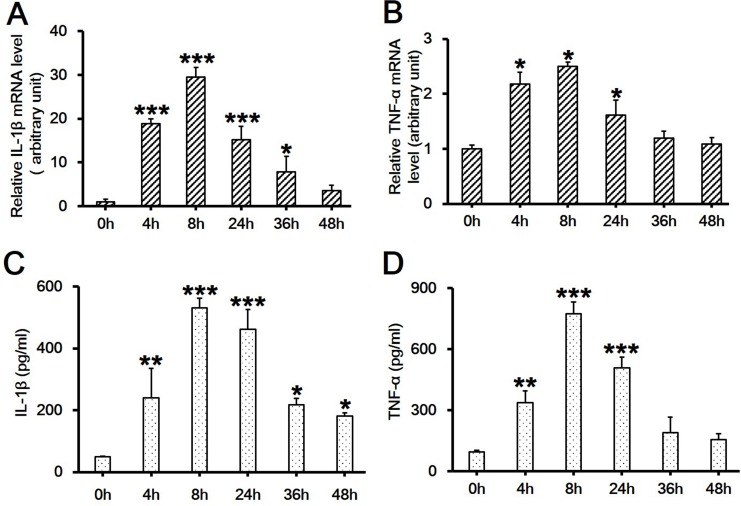
Assessment of the mRNA level and the release of IL-1β and TNF-α in RAW264.7 cell culture treated with 30 mM high glucose over time. (A-B) RAW264.7 cells were exposed to 30 mM D-glucose and cultured for indicated time periods of 0, 4, 8, 24, 36 and 48h. The mRNA levels of two inflammatory cytokines, IL-1β (A, *df* = 5, *f* = 62.812, *p* < 0.001) and TNF-α (B, *df* = 5, *f* = 5.492, *p* = 0.03), were assessed by real-time PCR. Each data point is the mean ± SEM of *n* = 3 and normalized against corresponding *GAPDH* mRNA level with the value at 0h arbitrarily set as 1. (C-D) The release of IL-1β (C, *df* = 5, *f* = 27.324, *p* < 0.001) and TNF-α (D, *df* = 5, *f* = 48.74, *p* < 0.001) in culture medium was assessed by ELISA. Each data point is the mean ± SEM of *n* = 3. *p < 0.05; **p < 0.01; ***p < 0.001.

It is known that tissue macrophages release cytokines that further activate the inflammatory program in neighboring adipocytes, exacerbating inflammation and insulin resistance[14]. It is also known that the proinflammatory cytokines, such as IL-1β and TNF-α, play key roles in the pathogenesis of several inflammatory diseases. Therefore, in order to elucidate whether high glucose would cause inflammatory response, we measured the mRNA levels of IL-1β and TNF-α in RAW264.7 cells stimulated with 30 mM D-glucose. Results showed that the mRNA levels of both IL-1β (Fig. 3A; *df* = 5, *f* = 62.812, *p* < 0.001) and TNF-α (Fig. 3B; *df* = 5, *f* = 5.492, *p* = 0.03) were significantly upregulated as early as 4 h post-high glucose treatment and peaked at 8 h. The tendency was maintained up to 48 h post-treatment for IL-1β (Fig. 3A) and 24 h for TNF-α (Fig. 3B). 30 mM D-glucose also induced more release of IL-1β up to 48 h (Fig. 3C; *df* = 5, *f* = 27.324, *p* < 0.001) and TNF-α up to 24 h (Fig. 3D; *df* = 5, *f* = 48.74, *p* < 0.001). The time course of the upregulation of cytokine levels showed great synchronization with the downregulation of SIRT1 levels in Fig. 2A-B.

### SIRT1 Mediates the Production and Secretion of Two Proinflammatory Cytokines Induced by High Glucose

**Fig 4:**
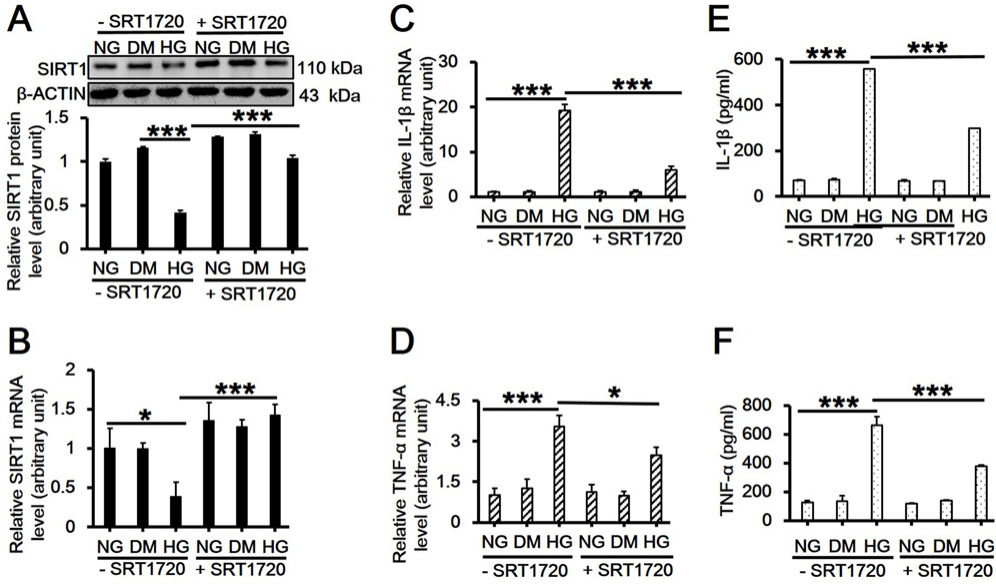
Effects of SIRT1 activator SRT1720 on the expression and/or release of SIRT1, IL-1β and TNF-α in RAW264.7 cell culture treated with 30 mM high glucose. (A-D) RAW264.7 cells were pretreated with 1 μM SRT1720 for 6h and then cultured in 30 mM D-glucose-containing medium for additional 8h. Cells were then harvested and used for western blot analysis for SIRT1protein level (A, *df* = 5, *f* = 53.307, *p* < 0.001), and real-time PCR analysis for *SIRT1* (B, *df* = 5, *f* = 16.042, *p* = 0.002), *IL-1β* (C, *df* = 5, *f* = 328.474, *p* < 0.001) or *TNF-α* mRNA level (D, *df* = 5, *f* = 43.581, *p* < 0.001). Each data point is the mean ± SEM of *n* = 3 and normalized against corresponding β-ACTIN protien level or *GAPDH* mRNA level with the value in NG without SRT1720 group arbitrarily set as 1. (E-F) The culture medium was also collected and used for ELISA for detecting the release of IL-1β (E, *df* = 5, *f* = 5739.982, *p* < 0.001) and TNF-α (F, *df* = 5, *f* = 108.365, *p* < 0.001). Each data point is the mean ± SEM of *n* = 3. *p < 0.05; ***p < 0.001. 30 mM of D-mannitol served as an osmotic control. ‘NG’, 5.6 mM normal glucose; ‘HG’, 30 mM high glucose; ‘DM’, 30 mM D-mannitol.

**Fig 5:**
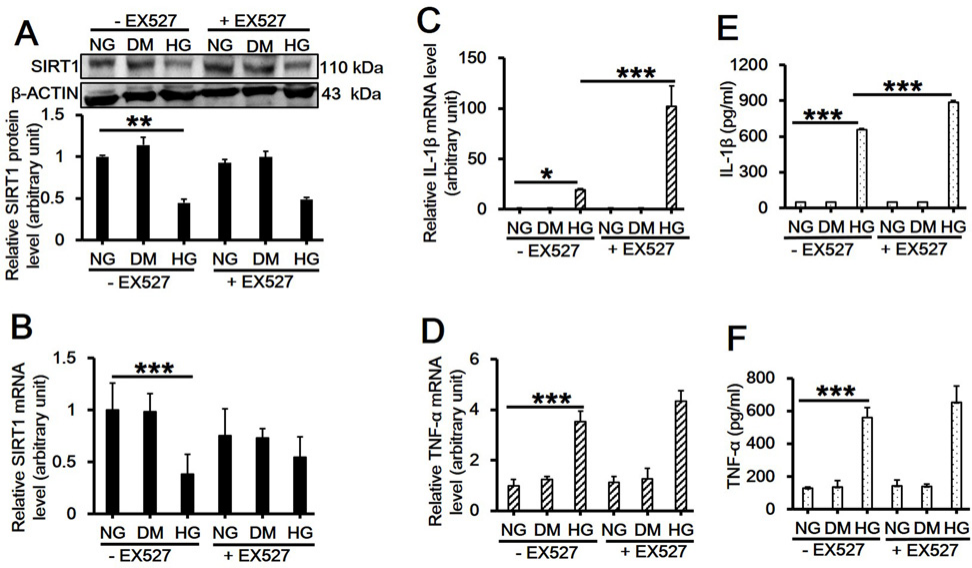
Effects of SIRT1 inhibitor EX527 on the expression and/or release of SIRT1, IL-1β and TNF-α in RAW264.7 cell culture treated with 30 mM high glucose. (A-D) RAW264.7 cells were pretreated with 10 μM EX527 for 6h and then cultured in 30 mM D-glucose-containing medium for additional 8h. Cells were then harvested and used for western blot analysis for SIRT1 protein level (A, *df* = 5, *f* = 6.194, *p* = 0.023), and real-time PCR analysis for *SIRT1* (B, *df* = 5, *f* = 7.118, *p* = 0.003), *IL-1β* (C, *df* = 5, *f* = 71.26, *p* < 0.001) or *TNF-*α mRNA level (D, *df* = 5, *f* = 50.638, *p* < 0.001). Each data point is the mean ± SEM of *n* = 3 and normalized against corresponding β-ACTIN protein level or *GAPDH* mRNA level with the value in NG without EX527 group arbitrarily set as 1. (E-F) The culture medium was also collected and used for ELISA for detecting the release of IL-1β (E, *df* = 5, *f* = 6057.757, *p* < 0.001) and TNF-α (F, *df* = 5, *f* = 47.79, *p* < 0.001). Each data point is the mean ± SEM of *n* = 3. *p < 0.05; **p < 0.01; ***p < 0.001. 30 mM of D-mannitol served as an osmotic control. ‘NG’, 5.6 mM normal glucose; ‘HG’, 30 mM high glucose; ‘DM’, 30 mM D-mannitol.

Previous reports have demonstrated that artificial overexpression of SIRT1 in hepatocytes led to the suppression of inflammatory response, whereas deletion of SIRT1 resulted in enhanced local inflammation [15,16]. In order to investigate the role of SIRT1 in inflammatory response induced by high glucose, RAW264.7 cells were pretreated with SRT1720, a putative SIRT1 activator, or EX527, its inhibitor, for 6 h and then received 30 mM D-glucose treatment for 8 h followed by immunoblotting and qPCR. Results showed that the reduced SIRT1 protein (Fig. 4A; *df* = 5, *f* = 53.307, *p* < 0.001; 5A; *df* = 5, *f* = 6.194, *p* = 0.023) or mRNA levels (Fig. 4B; *df* = 5, *f* = 16.042, *p* = 0.002; 5B; *df* = 5, *f* = 7.118, *p* = 0.003) by high glucose was significantly upregulated by SRT1720 (Fig. 4A-B), while had no further change with the use of EX527 (Fig. 5A-B). In the presence of SRT1720, high glucose-induced upregulation on the mRNA level and the release of IL-1β (Fig. 4C; *df* = 5, *f* = 328.474, *p* < 0.001; Fig. 4E; *df* = 5, *f* = 5739.982, *p* < 0.001) and TNF-α (Fig. 4D; *df* = 5, *f* = 43.581, *p* < 0.001; Fig. 4F; *df* = 5, *f* = 108.365, *p* < 0.001) were remarkably inhibited (Fig. 4C-F). Although EX527 did not further suppress the SIRT1 level, it did significantly increase IL-1β mRNA level (Fig. 5C; *df* = 5, *f* = 71.26, *p* < 0.001) and its release (Fig. 5E; *df* = 5, *f* = 6057.757, *p* < 0.001), and slightly upregulated TNF-α level (Fig. 5D; *df* = 5, *f* = 50.638, *p* < 0.001) and its release although showing no significant difference with high glucose treatment alone (Fig. 5F; *df* = 5, *f* = 47.79, *p* < 0.001).

### Loss of SIRT1 in Macrophages further Promotes the mRNA Level of Proinflammatory Cytokines Induced by High Glucose

**Fig 6:**
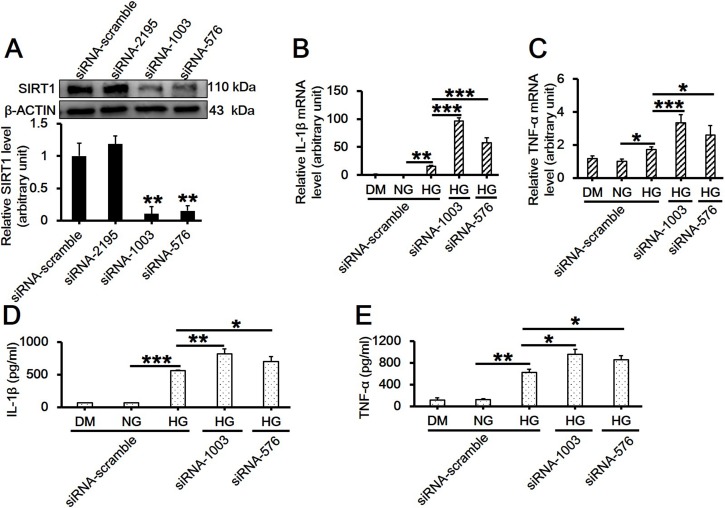
Effects of SIRT1 knockdown by RNAi on the mRNA level and the release of IL-1β and TNF-α in RAW264.7 cell culture treated with 30 mM high glucose. (A) Cells cultured in NG medium were transfected with 33 nM scramble or SIRT1-specific siRNA duplex (serial number: 2195, 1003, and 576) for 6h, incubated for another 42h, and then harvested for the detection of SIRT1 suppression by western blot (*df* = 3, *f* = 38.663, *p* = 0.002). (B-C) Cells receiving RNAi treatment were continued to be cultured in 30 mM D-glucose-containing medium for additional 8h, mRNA levels of IL-1β (B, *df* = 4, *f* = 250.541, *p* < 0.001) and TNF-α (C, *df* = 4, *f* = 24.145, *p* < 0.001) were assessed by real-time PCR. Each data point is the mean ± SEM of *n* = 3 and normalized against corresponding β-ACTIN protein level (A) or *GAPDH* mRNA level (B-C) with the value in NG with scramble siRNA group arbitrarily set as 1. (D-E) The culture medium was also collected and used for ELISA for detecting the release of IL-1β (D, *df* = 4, *f* = 110.68, *p* < 0.001) and TNF-α (E, *df* = 4, *f* = 80.041, *p* < 0.001). Each data point is the mean ± SEM of *n* = 3. *p < 0.05; **p < 0.01; ***p < 0.001. 30 mM of D-mannitol served as an osmotic control. ‘NG’, 5.6 mM normal glucose; ‘HG’, 30 mM high glucose; ‘DM’, 30 mM D-mannitol.

To further confirm the regulative role of SIRT1 in inflammatory response, RAW264.7 cells were subjected to RNAi followed by high glucose exposure. The mRNA levels of IL-1β and TNF-α were measured by real-time PCR, and their release were tested by ELISA. Results showed that both siRNA-1003 and siRNA-576 potently suppressed SIRT1 protein level (Fig. 6A; *df* = 3, *f* = 38.663, *p* = 0.002), and also significantly upregulated the mRNA level and the release of IL-1β (Fig. 6B; *df* = 4, *f* = 250.541, *p* < 0.001; 6D; *df* = 4, *f* = 110.68, *p* < 0.001) and TNF-α (Fig. 6C; *df* = 4, *f* = 24.145, *p* < 0.001; 6E; *df* = 4, *f* = 80.041, *p* < 0.001) in RAW264.7 cells treated with high glucose. This finding was highly consistent with the result using SIRT1 inhibitor EX527 as shown in Fig. 5C-F.

## Discussion

The pathogenesis of various inflammatory diseases such as sepsis is a complex process mediated by immune cells such as macrophages and monocytes [17,18]. Major efforts have been made to understand the proinflammatory process, especially focused on the early stage involving the gene expression of key proinflammatory mediators/cytokines [31]. Studies have shown that pretreatment of RAW264.7 macrophages with SRT1720 could downregulate LPS-induced release of proinflammatory factors, such as NO, PGE2, iNOS and COX-2, and proinflammatory cytokines, including TNF-α and IL-1β [19,20,21]. The progression of inflammatory diseases is usually associated with the irritability of high blood glucose [22]. Our study showed that high glucose significantly increased the production and secretion of IL-1β and TNF-α in RAW264.7 cells (Fig. 3A-D), which was consistent with a previous finding that high glucose increased the levels of TNF-α and IL-6 in the podocytes and thus induced subsequent inflammation and kidney fibrosis [23]. However, the signaling cascades mediating the effects of high glucose on the production of proinflammatory factors have not been clearly characterized.

It is well established that SIRT1 is a regulator of longevity and is associated with caloric restriction, energy metabolism, cell cycle and differentiation, however, its role in endotoxic or septic lethality has yet to be fully understood. There is accumulating evidence that SIRT1 could mediate the inhibition of resveratrol-induced release of proinflammatory cytokines [24,25,26]. A recent study has indicated that SIRT1 acted as a positive modulator in insulin signalings through PI3K in muscle cells [27,28]. Therefore, we hypothesized that SIRT1 might play a role in mediating high glucose-induced inflammatory response. In the present study, we first observed that high glucose significantly downregulated SIRT1 expression on both mRNA and protein levels in RAW264.7 cells in a dose- and time-dependent manner (Figs. 1B and 2A-B). Also, the concentration of D-glucose in culture medium did not change over time (Fig. 2C), suggesting the independence of SIRT1 reduction with extracellular glucose concentration. Next, we pretreated cells with SIRT1 activator SRT1720 or its inhibitor EX527 followed by high glucose exposure. Interestingly, SRT1720 abolished the downregulation of SIRT1 level induced by high glucose, and also suppressed the increased mRNA level and release of TNF-α and IL-1β (Fig. 4). On the contrary, EX527 exerted an inhibitory effect on SIRT1 activity without affecting SIRT1 expression on both mRNA and protein levels, which was consistent with the findings from Charles E. McCall’s lab [29,30], while it further upregulated the increased mRNA level and release of IL-1β induced by high glucose (Fig. 5). Importantly, results from the knockdown of SIRT1 by RNAi further confirmed above conclusion (Fig. 6). Our results showed a more than 120-fold increase on IL-1β mRNA level in SIRT1 RNAi cells under high glucose over that in scramble siRNA-transfected cells under normal glucose condition, this was consistent with a study showing that LPS increased IL-1β mRNA level more than 200 folds in SIRT1-knockdowned cells compared to that in untreated and scramble siRNA-transfected cells[31]. Therefore, SIRT1 appears to be a negative regulator for IL-1β at the early stage of inflammation. Taken together, our findings suggested a possible mechanism regulating the high glucose-induced inflammatory response in RAW264.7 cells, that is, high glucose inhibits SIRT1 expression, which subsequently stimulates the production of IL-1β and TNF-α and thus promotes inflammation process. However, the precise mechanism by which high glucose exerts inhibitory effects on SIRT1 expression is largely unknown. It is possible that high glucose may trigger a common signaling cascade that ultimately disturbs SIRT1 level [32,33].

While a number of studies have demonstrated that SIRT1 exhibits pronounced anti-inflammatory properties [34,35,36], the present study again provides evidences supporting its central role in the inhibition of high glucose-induced inflammatory response in RAW264.7 cells. To this end, our study attempts to reveal, at least partly, the molecular mechanism of high glucose-induced macrophage activation, which is desirable in delineating the molecular therapeutic target to reduce inflammatory response in various inflammation-related diseases such as sepsis. Nonetheless, it is noteworthy that our findings were mainly based on an *in vitro* high glucose model, which cannot fully simulate the *in vivo* condition in sepsis. Moreover, the precise relationship between the high glucose-induced inflammation and SIRT1 expression has not been extensively investigated. Therefore, further studies are needed to thoroughly understand the mechanism on high glucose-induced inflammatory response in sepsis.
