# Correction: SIRT1 Is a Regulator in High Glucose-Induced Inflammatory Response in RAW264.7 Cells

**DOI:** 10.1371/journal.pone.0127605

**Published:** 2015-05-04

**Authors:** The PLOS ONE Staff

The first four authors, Yanhui Jia, Zhao Zheng, Yunchuan Wang, and Qin Zhou, should be noted as contributing equally to this work. Weixia Cai is not included in this group of equal contributors.
